# Predictive value of ^18^F‐FDG PET/CT and serum tumor markers for tumor mutational burden in patients with non‐small cell lung cancer

**DOI:** 10.1002/cam4.6665

**Published:** 2023-11-15

**Authors:** Qian Zhang, Xiuli Tao, Pei Yuan, Zewei Zhang, Jianming Ying, Lei Guo, Ning Li, Shuhang Wang, Jing Li, Ying Liu, Wei Guo, Shijun Zhao, Ning Wu

**Affiliations:** ^1^ Department of Diagnostic Radiology, National Cancer Center/National Clinical Research Center for Cancer/Cancer Hospital Chinese Academy of Medical Sciences and Peking Union Medical College Beijing China; ^2^ Department of Nuclear Medicine (PET‐CT Center) National Cancer Center/National Clinical Research Center for Cancer/Cancer Hospital, Chinese Academy of Medical Sciences and Peking Union Medical College Beijing China; ^3^ Department of Pathology, National Cancer Center/National Clinical Research Center for Cancer/Cancer Hospital Chinese Academy of Medical Sciences and Peking Union Medical College Beijing China; ^4^ Department of Clinical Trial Center, National Cancer Center/National Clinical Research Center for Cancer/Cancer Hospital Chinese Academy of Medical Sciences and Peking Union Medical College Beijing China; ^5^ Department of Thoracic Surgery, National Cancer Center/National Clinical Research Center for Cancer/Cancer Hospital Chinese Academy of Medical Sciences and Peking Union Medical College Beijing China

**Keywords:** ^18^F‐FDG PET/CT, immunotherapy, non‐small cell lung cancer, serum tumor marker, tumor mutational burden

## Abstract

**Purpose:**

To investigate the correlations between metabolic parameters (MPs) of ^18^F‐fluorodeoxyglucose (FDG) uptake on positron emission tomography/computed tomography (PET/CT), serum tumor markers (STMs), and tumor mutational burden (TMB) in patients with non‐small cell lung cancer (NSCLC).

**Materials and Methods:**

In this retrospective study, we enrolled 129 patients with NSCLC (males, 78; females, 51) who underwent baseline TMB and STM tests and ^18^F‐FDG PET/CT scans before treatment between March 2018 and September 2022. Patients were categorized into TMB‐high (TMB ≥10 mutations/Mb; *n* = 27 [20.9%]) and non‐TMB‐high (TMB <10 mutations/Mb; *n* = 102 [79.1%]) groups. Binary logistic regression analyses were performed to determine independent predictors of TMB‐high. Univariate and multivariate linear regression analyses were performed to determine independent predictors of TMB level on a log scale. Subgroup analyses for adenocarcinoma (ADC), ADC with *EGFR*+, ADC with *EGFR*−, and squamous cell carcinoma (SCC) were performed.

**Results:**

For ADC, all MPs (SUL_peak_, SUL_max_, SUL_mean_, MTV, and TLG) were significantly higher in the TMB‐high group than the non‐TMB‐high group; smoker (odds ratio [OR] = 27.08, *p* = 0.018), *EGFR*+ (OR = 0.03, *p* = 0.033), *KRAS*+ (OR = 7.98, *p* = 0.083), high CEA (OR = 33.56, *p* = 0.029), and high CA125 (OR = 13.68, *p* = 0.030) were independent predictors of TMB‐high; and all MPs showed significant positive linear correlations with TMB on a log scale, with SUL_peak_ as an independent predictor. However, no significant correlation was observed for SCC.

**Conclusion:**

MPs and STMs can predict the TMB level for patients with ADC, and may serve as potential substitutes for TMB with increased value and easy implementation in guiding immunotherapy through noninvasive methods.

## 1 INTRODUCTION

Immune checkpoint inhibitor (ICI) therapy has revolutionized the treatment of advanced non‐small cell lung cancer (NSCLC). Unfortunately, the response rate to ICI monotherapy is only 15%–20% for unselected patients.^1^, ^2^ Predictive biomarkers remain a major challenge for clinical applications. Tumor mutational burden (TMB), defined as the total number of somatic mutations per coding area of a tumor genome, has been acknowledged as a promising biomarker of response to ICIs. A higher TMB is associated with higher response rates and longer progression‐free survival.^3^ However, TMB has not been widely available clinically because of difficulties in detection, including invasive procedures followed by pathological assessment, the inability to obtain sufficient specimens sometimes or obtain specimens repeatedly, and expensive detection methods for next‐generation sequencing. Therefore, it is important to explore noninvasive surrogate biomarkers for TMB.

As a functional noninvasive imaging modality based on glucose metabolism, ^18^F‐fluorodeoxyglucose (FDG) positron emission tomography/computed tomography (PET/CT) is a significant imaging modality. Previous studies have reported that baseline metabolic parameters (MPs) of NSCLC may be predictive factors for response to immunotherapy.^4^ Serum tumor markers (STMs) from peripheral blood have the advantages of being noninvasive, convenient, and inexpensive and are potentially useful in the early diagnosis, treatment monitoring, and prognosis evaluation of NSCLC.^5^, ^6^ STMs can serve as ideal tumor‐associated antigens for inducing effective tumor immunity.^7^ Some retrospective analyses have also demonstrated that pretreated STMs are prognostic predictors of immunotherapy.^8^, ^9^ Therefore, we hypothesized that there may be links between MPs, STMs, and TMB.

To date, few studies have explored the correlations between MPs, STMs, and TMB,^10^, ^11^, ^12^ and the results remain controversial. Haghighat‐Jahromi et al. confirmed an independent correlation between the maximum standardized uptake value (SUV_max_) and TMB in various tumor types.^10^ Ono et al. reported that SUV_max_ and carcinoembryonic antigen (CEA) were independent predictors of TMB in NSCLC.^12^ However, Moon et al. found no significant relationship between metabolic features and TMB in lung cancer.^11^ Additionally, these studies had some limitations: (a) subgroup analyses of pathological type and driver gene mutation type were not performed; (b) the time interval of PET/CT scan, STM test, and biopsy/surgery were not determined; (c) the cutoff of TMB‐high was different; and (d) limited MPs and STMs were studied.

To complement the deficiency in the extant literature, we aimed to further explore the correlations of MPs and STMs with TMB. Detailed analyses of different pathological types and driver gene mutation types were performed. We expect to provide new insights for potential substitutes to TMB which has clinical utility.

## 2 MATERIALS AND METHODS

### 2.1 Patient selection

We enrolled 2597 patients with lung cancer who underwent PET/CT examinations with data retrieved from the PET/CT Center data system between March 2018 and September 2022. The study was conducted according to the guidelines of the Declaration of Helsinki and approved by the Institutional Review Board of the Institutional Ethics Committee of our hospital. The inclusion criteria were as follows: (a) pathologically confirmed NSCLC; (b) baseline TMB data of the primary tumor tissue on biopsy/surgery samples obtained before treatment, excluding TMB data of metastases and lymph nodes; (c) baseline PET/CT scan performed before treatment; (d) time interval between biopsy/surgery and PET/CT scan was within 1 month to ensure an acceptable temporal correlation between imaging and genomic evaluation; and (e) measurable and describable tumor lesions on PET/CT. When a patient had multiple primary lung cancers, the lesion undergoing biopsy/surgery for TMB test was included in the analyses.

Finally, 129 patients who underwent TMB tests and PET/CT scans before treatment were enrolled in the study. Clinical and demographic characteristics were retrospectively collected from medical records. We further collected baseline STM data of the enrolled patients, and the time interval between biopsy/surgery and the STM test was within 1 month. Ultimately, 98 of the 129 enrolled patients had available STM data.

### 2.2 FDG PET/CT acquisition

All patients underwent a whole‐body PET/CT scan as per the standard protocol of our hospital. Head‐to‐groin imaging was performed using an integrated PET/CT device (Discovery 690; GE Healthcare). Patients were asked to fast for at least 6 h before the scan, and blood glucose level (normal, <145 mg/dL) was determined. Patients were intravenously injected with ^18^F‐FDG at a dose of 3.70–4.44 MBq/kg. Three‐dimensional (3D) whole‐body PET/CT scans with CT were acquired approximately 50–70 min after ^18^F‐FDG injection. PET images were obtained in 3D mode at a rate of 2 min per frame from head to groin (generally, 7–8 beds location) and reconstructed using the VPFX‐S algorithm, with two iterations, 24 subsets, and a 4 mm Gaussian postfilter. Spiral CT scans were performed using a standardized protocol (tube voltage, 120 kV; tube current, 150 mA; slice thickness, 3.75 mm; and rotation speed, 0.5 s) without intravenous contrast administration. All patients underwent a breath‐hold thoracic spiral CT scan at 120 kV, 205 mA, 0.5 s per tube rotation, and a thickness of 5 mm after the PET/CT scan. Images were reconstructed from a thickness of 1.25 mm and 0.8 mm space.

### 2.3 Image analysis

PET images were analyzed using PETVCAR (PET Volume Computerized Assisted Reporting), an automated segmentation software system on an Advantage Workstation (version 4.6; GE Healthcare). The volume of interest (VOI) of the whole tumor was autocontoured and segmented using a 3D cube. The VOI was placed by one radiologist (Q.Z.) and verified by another experienced radiologist (X.L.T.) to ensure that the 3D cube contained all FDG PET‐positive areas of the primary tumor in the axial, coronal, and sagittal planes. Disagreements were resolved by consultation. The two radiologists were blinded to the clinical data of all patients when placing the VOI.

PETVCAR is an automated segmentation software system that uses an iterative adaptive algorithm to detect the threshold level. It separates the target volume from the background tissue by weighting SUV_max_ and SUV_mean_ within the target volume with a weighting factor of 0.5.^13^ PETVCAR was used to calculate the following parameters of the primary tumor: SUL_max_, SUL_mean_, SUL_peak_, metabolic tumor volume (MTV), and total lesion glycolysis (TLG). SUL_max_ was defined as the maximum SUL derived from the single voxel with the highest uptake; SUL_mean_ was calculated as the sum of SUL in each voxel divided by the number of voxels within the target volume; SUL_peak_ was defined as the mean SUL value measured in a 1 cm^3^ volume spherical region of interest centered around the hottest point of the tumor; MTV represented the volume of the contoured tumor tissue with active FDG uptake; and TLG was obtained by multiplying SUL_mean_ by MTV.

### 2.4 Serum tumor markers assessment

Based on clinical applications, STMs, including CEA, carbohydrate antigen 125 (CA125), cytokeratin 19 fragment (Cyfra21‐1), and squamous cell‐associated antigen (SCC‐Ag), were tested using immunoelectrochemistry according to institutional standard laboratory procedures. Upper limits of normal were 5.0 ng/mL for CEA, 35.0 U/mL for CA125, 3.3 ng/mL for Cyfra21‐1, and 1.5 ng/mL for SCC‐Ag, respectively. Records were obtained from the database and categorized as high or normal based on the upper limits.

### 2.5 Histologic and genomic analysis

The approach used for obtaining pathological specimens was determined according to the patient's condition: 26 patients underwent surgery, 67 underwent CT‐guided lung biopsy, and 36 underwent bronchofibroscopy biopsy. Next‐generation sequencing was used to detect the TMB level, epidermal growth factor receptor (*EGFR*) mutation status, and Kirsten rat sarcoma viral oncogene homolog (*KRAS*) mutation status. Records were obtained from the official reports of the Pathology Department at our hospital. TMB was measured as mutations per megabase pair. Patients were categorized into TMB‐high (≥10 mutations/Mb) and non‐TMB‐high (<10 mutations/Mb) groups. Currently, there is no generally accepted cutoff value to define TMB‐high. In this study, TMB‐high was defined as ≥10 mutations/Mb as this category is consistent with those used in several randomized trials in lung cancer^3^, ^14^, ^15^ and adopted by the Food and Drug Administration for refractory solid tumor approval of anti–PD‐1 therapy.^16^ In addition, it is also the minimum threshold developed by a multistakeholder group convened by the Friends of Cancer Research to coordinate a standard cutoff across clinical trials.^17^, ^18^

### 2.6 Statistical analysis

Descriptive statistics were used to analyze the differences between the TMB‐high and non‐TMB‐high groups. Categorical data were presented as numbers (percentages). The Kolmogorov–Smirnov test was used to analyze the distribution normality of continuous data. Normally distributed data were presented as mean ± standard deviation, whereas non‐normally distributed data were presented as median (range). Comparisons between the TMB‐high and non‐TMB‐high groups were evaluated using Pearson's chi‐squared test or Fisher's exact test for categorical variables and independent samples *t*‐test or Mann–Whitney U test for continuous variables. Binary logistic regression analyses with backward stepwise selection were performed to determine independent predictors of TMB‐high. Univariate and multivariate linear regression analyses were performed to determine the correlative factors of log_10_‐transformed TMB, denoted as TMB (lg). TMB, MTV, and TLG were required to transform into the shifted log scale in the linear regression analyses because of the highly skewed distribution. Significant variables in the univariate linear regression were included in the multivariate linear regression model with backward stepwise selection.

Notably, there was significant collinearity among PET parameters. Significant explanatory variables in multivariate linear regression analyses were identified using a backward stepwise elimination method. Variables with collinearity were not retained. The final regression model was checked to make sure each variable made a significant contribution. Multicollinearity analyses showed that the variance inflation factors for all enrolled variables were below 5.0. Finally, through model selection, three variables (SUL_max_, SUL_mean_, and TLG) were excluded, and two variables (SUL_peak_ and MTV) were included to construct the multivariate linear regression analyses.

Subgroup analyses for adenocarcinoma (ADC), ADC with *EGFR*+, ADC with *EGFR*−, and squamous cell carcinoma (SCC) were performed. Statistical analyses were conducted using SPSS (version 25.0; IBM Corp.). Statistical significance was set at *p* < 0.05.

## 3 RESULTS

### 3.1 Patient characteristics

Based on the inclusion criteria, 129 patients with NSCLC, all of whom had baseline TMB and PET/CT scans before treatment, were enrolled in the present study. Among the 129 enrolled patients (78 males and 51 females; age, 61 ± 10 years), 76.0% (98/129) had ADC and 24.0% (31/129) had SCC. Regarding ADC, 54.1% (53/98) were *EGFR*+ and 45.9% (45/98) were *EGFR*−. Patients in the TMB‐high group accounted for 20.9% (27/129) and those in the non‐TMB‐high group accounted for 79.1% (102/129), with a median TMB of 13.0 and 3.2 mutations/Mb, respectively. The values of the MPs were 8.3 ± 4.1, 11.1 ± 5.1, 5.2 ± 2.3, 15.8 (0.4–238.0), and 75.7 (0.7–2052.5) for SUL_peak_, SUL_max_, SUL_mean_, MTV, and TLG, respectively. Ninety‐eight of the 129 enrolled patients had available STM data. The percentage of high STMs was 45.9% (45/98), 29.6% (29/98), 56.1% (55/98), and 17.3% (17/98) for CEA, CA125, Cyfra21‐1, and SCC‐Ag, respectively. Clinical information is summarized in Tables 1 and 2.

**TABLE 1:** Clinicopathological characteristics of NSCLC.

	All patients	Non‐TMB‐High	TMB‐High	*p* Value
Sex				
Male	78	55 (70.5)	23 (29.5)	0.003
Female	51	47 (92.2)	4 (7.8)	
Age (years)	61 ± 10	61 ± 10	64 ± 8	0.146
Histological subtype				
ADC	98	84 (85.7)	14 (14.3)	0.001
SCC	31	18 (58.1)	13 (41.9)	
Clinical stage				
I or II	40	32 (80.0)	8 (20.0)	0.862
III or IV	89	70 (78.7)	19 (21.3)	
Smoking status				
Smoker	61	40 (65.6)	21 (34.4)	<0.001
Nonsmoker	68	62 (91.2)	6 (8.8)	
History of malignancy				
Yes	36	25 (69.4)	11 (30.6)	0.095
No	93	77 (82.8)	16 (17.2)	
ECOG PS				
0–1	116	92 (79.3)	24 (20.7)	1.000
2	13	10 (76.9)	3 (23.1)	
*EGFR* mutation				
*EGFR*+	54	53 (98.1)	1 (1.9)	<0.001
*EGFR*−	75	49 (65.3)	26 (34.7)	
*KRAS* mutation				
*KRAS*+	18	11 (61.1)	7 (38.9)	0.088
*KRAS*−	111	91 (82.0)	20 (18.0)	
CEA				
High	45	34 (75.6)	11 (24.4)	0.503
Normal	53	43 (81.1)	10 (18.9)	
CA125				
High	29	19 (65.5)	10 (34.5)	0.041
Normal	69	58 (84.1)	11 (15.9)	
Cyfra21‐1				
High	55	39 (70.9)	16 (29.1)	0.037
Normal	43	38 (88.4)	5 (11.6)	
SCC‐Ag				
High	17	9 (52.9)	8 (47.1)	0.012
Normal	81	68 (84.0)	13 (16.0)	

Abbreviations: ADC, adenocarcinoma; CA125, carbohydrate antigen 125; CEA, carcinoembryonic antigen; Cyfra21‐1, cytokeratin 19 fragment; ECOG PS, Eastern Cooperative Oncology Group Performance Status; *EGFR*, epidermal growth factor receptor; *KRAS*, Kirsten rat sarcoma viral oncogene homolog; NSCLC, non‐small cell lung cancer; SCC, squamous cell carcinoma; SCC‐Ag, squamous cell‐associated antigen; TMB, tumor mutational burden.

**TABLE 2:** PET parameters of NSCLC.

	All patients	Non‐TMB‐High	TMB‐High	*p* Value
SUL_peak_	8.3 ± 4.1	7.9 ± 4.0	10.4 ± 3.8	0.003
SUL_max_	11.1 ± 5.1	10.4 ± 5.0	13.4 ± 4.7	0.006
SUL_mean_	5.2 ± 2.3	5.0 ± 2.3	6.2 ± 2.0	0.016
MTV	15.8 (0.4–238.0)	13.2 (0.4–222.0)	32.7 (1.7–238.0)	<0.001
TLG	75.7 (0.7–2052.5)	58.7 (0.7–2052.5)	205.8 (7.7–1366.8)	<0.001

Abbreviations: MTV, metabolic tumor volume; NSCLC, non‐small cell lung cancer; PET, positron emission tomography; SUL, the standardized uptake value corrected by lean body mass; TLG, total lesion glycolysis; TMB, tumor mutational burden.

**TABLE 3:** Clinicopathological characteristics of ADC, ADC with *EGFR*−, and SCC^a^.

	ADC			ADC with *EGFR*−			SCC		
	Non‐TMB‐High	TMB‐High	*p Value*	Non‐TMB‐High	TMB‐High	*p Value*	Non‐TMB‐High	TMB‐High	*p Value*
Sex									
Male	37	11	0.017	20	10	0.492	18	12	0.419
Female	47	3		12	3		0	1	
Age (years)	60 ± 11	65 ± 7	0.130	59 ± 12	65 ± 7	0.067	62 ± 6	62 ± 9	0.922
Clinical stage									
I or II	24	4	1.000	5	4	0.411	10	9	0.484
III or IV	60	10		27	9		8	4	
Smoking status									
Smoker	22	10	0.002	15	9	0.205	18	11	0.168
Nonsmoker	62	4		17	4		0	2	
History of malignancy									
Yes	21	6	0.288	8	5	0.473	4	5	0.433
No	63	8		24	8		14	8	
ECOG PS									
0–1	74	11	0.584	29	10	0.334	18	13	NA
2	10	3		3	3		0	0	
*EGFR* mutation									
*EGFR*+	52	1	<0.001	0	0	NA	1	0	1.000
*EGFR*−	32	13		32	13		17	13	
*KRAS* mutation									
*KRAS*+	10	7	0.002	10	6	0.494	1	0	1.000
*KRAS*−	74	7		22	7		17	13	
CEA									
High	28	9	0.047	9	8	0.141	6	2	0.380
Normal	36	3		13	3		7	7	
CA125									
High	16	8	0.012	8	7	0.163	3	2	1.000
Normal	48	4		14	4		10	7	
Cyfra21‐1									
High	33	10	0.042	13	9	0.258	6	6	0.415
Normal	31	2		9	2		7	3	
SCC‐Ag									
High	5	1	1.000	2	1	1.000	4	7	0.080
Normal	59	11		20	10		9	2	

^a^ The subgroup analysis of ADC with *EGFR*+ was not performed because only one patient had TMB‐high in this subgroup.

Abbreviations: ADC, adenocarcinoma; CA125, carbohydrate antigen 125; CEA, carcinoembryonic antigen; Cyfra21‐1, cytokeratin 19 fragment; ECOG PS, Eastern Cooperative Oncology Group Performance Status; *EGFR*, epidermal growth factor receptor; *KRAS*, Kirsten rat sarcoma viral oncogene homolog; SCC, squamous cell carcinoma; SCC‐Ag, squamous cell‐associated antigen; TMB, tumor mutational burden.

### 3.2 Correlations of MPs and STMs with TMB‐high

Tables 1, 2, 3, 4 demonstrate the differences between the TMB‐high and non‐TMB‐high groups for NSCLC, ADC, ADC with *EGFR*−, and SCC, respectively. Subgroup analysis of ADC with *EGFR*+ was not performed because only one patient had TMB‐high in this subgroup.

**TABLE 4:** PET parameters of ADC, ADC with *EGFR−*, and SCC^a^.

	ADC			ADC with *EGFR*−			SCC		
	Non‐TMB‐High	TMB‐High	*p* Value	Non‐TMB‐High	TMB‐High	*p* Value	Non‐TMB‐High	TMB‐High	*p* Value
SUL_peak_	7.3 ± 3.9	9.9 ± 3.1	0.020	8.5 ± 3.6	9.6 ± 2.9	0.376	10.0 ± 3.8	10.9 ± 4.5	0.539
SUL_max_	9.8 ± 4.9	12.8 ± 4.0	0.036	11.2 ± 4.4	12.4 ± 3.9	0.397	13.2 ± 4.9	14.1 ± 5.4	0.608
SUL_mean_	4.7 ± 2.2	6.0 ± 1.9	0.048	5.3 ± 2.1	5.8 ± 1.9	0.484	6.4 ± 1.9	6.4 ± 2.2	0.998
MTV	11.2 (0.4–200.0)	36.8 (4.5–238.0)	0.001	21.9 (0.4–200.0)	40.8 (4.5–238.0)	0.052	25.1 (2.0–222.0)	24.5 (1.7–149.0)	0.567
TLG	49.2 (0.7–2052.5)	231.3 (20.4–1366.8)	<0.001	105.1 (0.7–2052.5)	256.9 (20.4–1366.8)	0.048	146.3 (8.9–1543.8)	102.6 (7.7–1208.9)	0.798

^a^ The subgroup analysis of ADC with *EGFR*+ was not performed because only one patient had TMB‐high in this subgroup.

Abbreviations: ADC, adenocarcinoma; *EGFR*, epidermal growth factor receptor; MTV, metabolic tumor volume; PET, positron emission tomography; SCC, squamous cell carcinoma; SUL, the standardized uptake value corrected by lean body mass; TLG, total lesion glycolysis; TMB, tumor mutational burden.

TMB‐high was associated with male, SCC, smoker, and *EGFR*− for NSCLC, and male, smoker, *EGFR*−, and *KRAS*+ for ADC (all *p* < 0.05). TMB‐high was additionally associated with high CA125, Cyfra21‐1, and SCC‐Ag for NSCLC, and high CEA, CA125, and Cyfra21‐1 for ADC (all *p* < 0.05). All MPs (SUL_peak_, SUL_max_, SUL_mean_, MTV, and TLG) were significantly higher in the TMB‐high group than in the non‐TMB‐high group for NSCLC and ADC (all *p* < 0.05). In the subgroup analysis of ADC with *EGFR*−, TLG (*p* = 0.048) was significantly higher in the TMB‐high group than in the non‐TMB‐high group. However, no significant correlation was observed for SCC.

**FIGURE 1:**
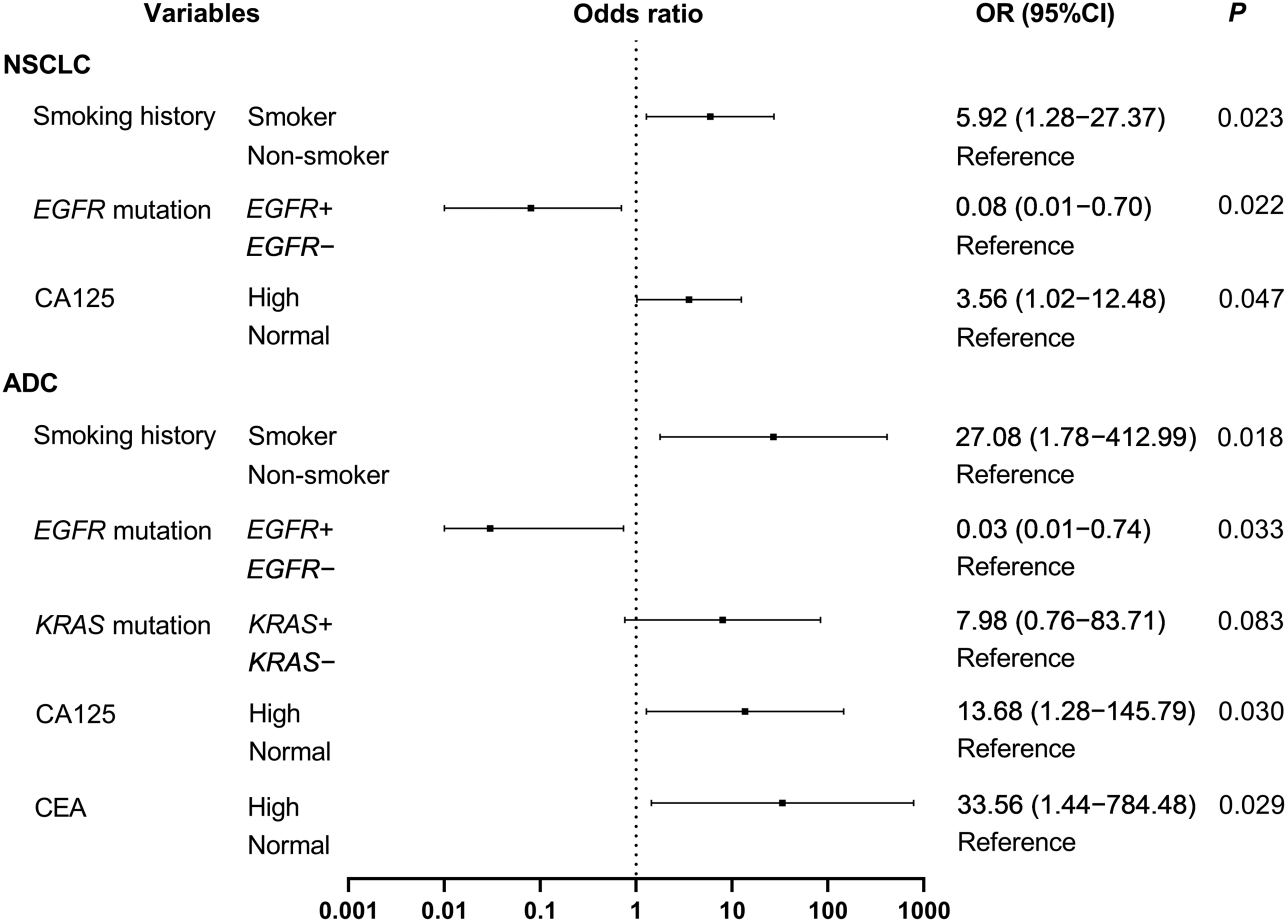
Independent predictors of TMB‐high by binary logistic regression analysis for NSCLC and ADC. TMB, tumor mutational burden; NSCLC, non‐small cell lung cancer; ADC, adenocarcinoma; SCC, squamous cell carcinoma; *EGFR*, epidermal growth factor receptor; *KRAS*, Kirsten rat sarcoma viral oncogene homolog; CEA, carcinoembryonic antigen; CA125, carbohydrate antigen 125; SUL, standardized uptake value corrected by lean body mass.

The results of the binary logistic regression analyses for NSCLC and ADC are shown in Figure 1. Smoker (odds ratio [OR] = 5.92, *p* = 0.023), *EGFR*+ (OR = 0.08, *p* = 0.022), and high CA125 (OR = 3.56, *p* = 0.047) were independent predictors of TMB‐high for NSCLC, and smoker (OR = 27.08, *p* = 0.018), *EGFR*+ (OR = 0.03, *p* = 0.033), *KRAS*+ (OR = 7.98, *p* = 0.083), high CEA (OR = 33.56, *p* = 0.029), and high CA125 (OR = 13.68, *p* = 0.030) were independent predictors of TMB‐high for ADC.

### 3.3 Correlations of MPs and STMs with TMB on a log scale

Owing to the highly skewed distribution of TMB, linear regression analysis was used to identify independent predictors of TMB on a log scale. Table 5 demonstrates the univariate linear regression analyses for NSCLC, ADC, ADC with *EGFR*+, ADC with *EGFR*−, and SCC, respectively. All MPs showed significant positive linear correlations with TMB (lg) for NSCLC, ADC, ADC with *EGFR*+, and ADC with *EGFR*− (all *p* < 0.05). However, no significant correlation was observed for SCC.

**TABLE 5:** Univariate linear regression analysis of the relationship with TMB on a log scale for NSCLC, ADC, ADC with *EGFR*+, ADC with *EGFR*−, and SCC.

	NSCLC		ADC		ADC with *EGFR*+		ADC with *EGFR*−		SCC	
	*β*	*p* Value	*β*	*p* Value	*β*	*p* Value	*β*	*p* Value	*β*	*p* Value
Age	0.238	0.007	0.253	0.012	0.190	0.172	0.330	0.027	0.104	0.577
Sex (Male/Female)	0.347	<0.001	0.219	0.030	0.015	0.916	0.252	0.095	−0.147	0.429
Histological subtype (ADC/SCC)	−0.447	<0.001	NA	NA	NA	NA	NA	NA	NA	NA
Clinical stage (III‐IV/I‐II)	0.104	0.240	0.183	0.072	0.214	0.124	0.085	0.580	0.098	0.601
Smoking status (Smoker/Nonsmoker)	0.406	<0.001	0.264	0.009	0.026	0.853	0.260	0.085	−0.187	0.313
History of malignancy (Yes/No)	0.077	0.384	0.075	0.463	−0.114	0.415	0.216	0.153	0.116	0.534
ECOG PS (0–1/2)	0.025	0.781	0.130	0.203	0.058	0.682	0.200	0.187	NA	NA
*EGFR* mutation *(EGFR*+/*EGFR*−)	−0.416	<0.001	−0.272	0.007	NA	NA	NA	NA	−0.309	0.091
*KRAS* mutation *(KRAS*+/*KRAS*−)	0.152	0.085	0.281	0.005	0.344	0.012	0.164	0.282	0.026	0.889
CEA (High/Normal)	0.178	0.079	0.294	0.010	0.199	0.202	0.396	0.023	−0.125	0.580
CA125 (High/Normal)	0.161	0.113	0.251	0.029	0.163	0.296	0.185	0.302	−0.082	0.715
Cyfra21‐1 (High/Normal)	0.200	0.048	0.210	0.069	0.120	0.443	0.204	0.255	0.333	0.130
SCC‐Ag (High/Normal)	0.201	0.047	−0.037	0.753	−0.272	0.077	0.127	0.483	0.301	0.173
SUL_peak_	0.449	<0.001	0.461	<0.001	0.411	0.002	0.455	0.002	−0.024	0.898
SUL_max_	0.437	<0.001	0.445	<0.001	0.393	0.004	0.448	0.002	−0.032	0.864
SUL_mean_	0.427	<0.001	0.424	<0.001	0.380	0.005	0.410	0.005	−0.101	0.589
MTV (lg)	0.379	<0.001	0.379	<0.001	0.291	0.034	0.318	0.033	0.080	0.669
TLG (lg)	0.436	<0.001	0.433	<0.001	0.348	0.011	0.400	0.007	0.034	0.858

Abbreviations: ADC, adenocarcinoma; CA125, carbohydrate antigen 125; CEA, carcinoembryonic antigen; Cyfra21‐1, cytokeratin 19 fragment; ECOG PS, Eastern Cooperative Oncology Group Performance Status; *EGFR*, epidermal growth factor receptor; *KRAS*, Kirsten rat sarcoma viral oncogene homolog; MTV, metabolic tumor volume; NA, not applicable; NSCLC, non‐small cell lung cancer; SCC, squamous cell carcinoma; SCC‐Ag, squamous cell‐associated antigen; SUL, the standardized uptake value corrected by lean body mass; TLG, total lesion glycolysis; TMB, tumor mutational burden.

**FIGURE 2:**
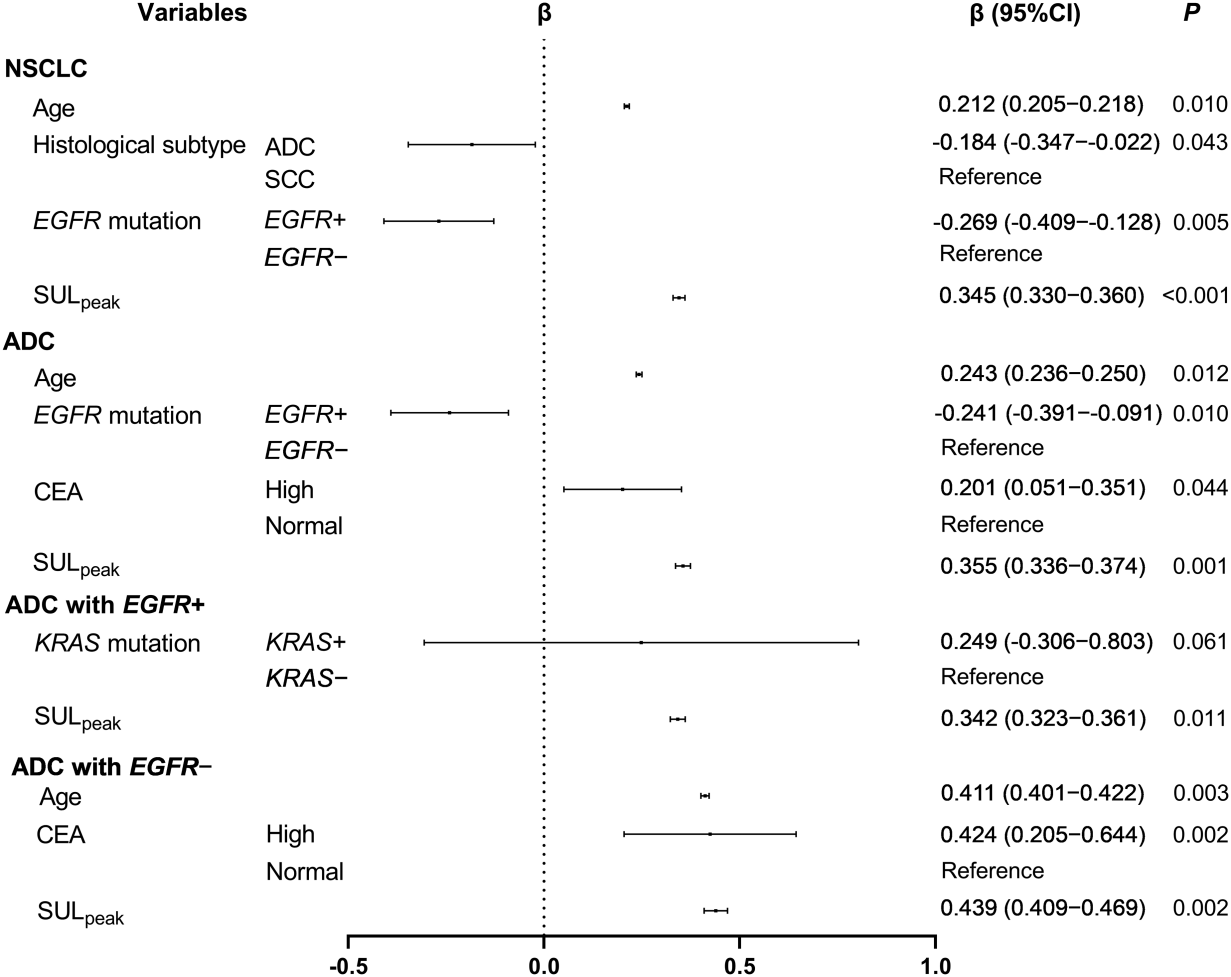
Independent predictors of TMB on a log scale by multivariate linear regression analysis for NSCLC, ADC, ADC with *EGFR*+, and ADC with *EGFR*−. TMB, tumor mutational burden; NSCLC, non‐small cell lung cancer; ADC, adenocarcinoma; SCC, squamous cell carcinoma; *EGFR*, epidermal growth factor receptor; *KRAS*, Kirsten rat sarcoma viral oncogene homolog; CEA, carcinoembryonic antigen; SUL, standardized uptake value corrected by lean body mass.

**FIGURE 3:**
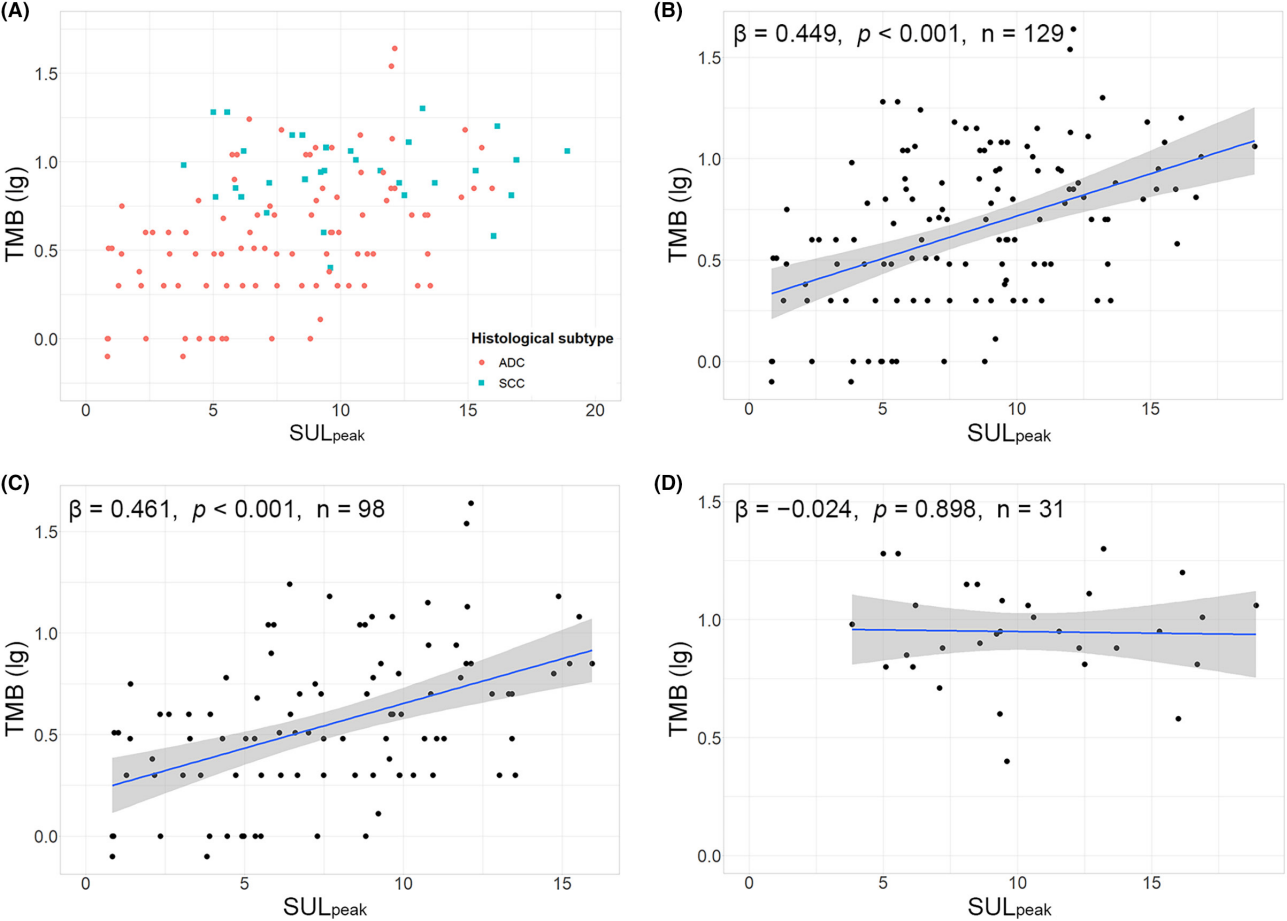
Correlations between SUL_peak_ and TMB for NSCLC, ADC, and SCC on a log scale. (A) Scatter diagram showing the distribution of SUL_peak_ and TMB (lg) for ADC and SCC. (B,C) Positive correlations between SUL_peak_ and TMB (lg) for NSCLC (B) and ADC (C). (D) No significant correlation between SUL_peak_ and TMB (lg) for SCC. TMB, tumor mutational burden; NSCLC, non‐small cell lung cancer; SUL, standardized uptake value corrected by lean body mass; ADC, adenocarcinoma; SCC, squamous cell carcinoma.

**FIGURE 4:**
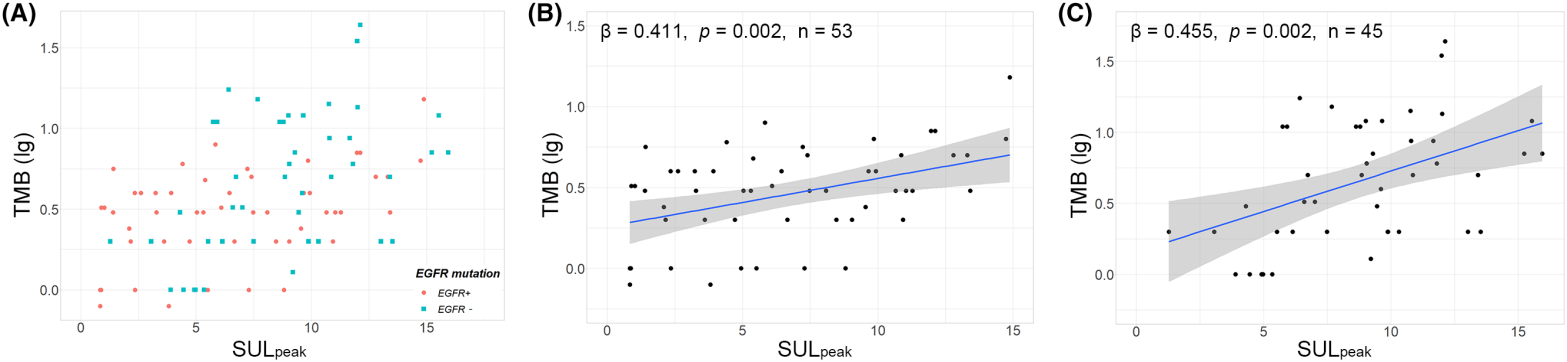
Correlations between SUL_peak_ and TMB for ADC with *EGFR*+ and *EGFR*− on a log scale. (A) Scatter diagram showing the distribution of SUL_peak_ and TMB (lg) for ADC with *EGFR*+ and *EGFR*−. (B,C) Positive correlations between SUL_peak_ and TMB (lg) for ADC with *EGFR*+ (B) and ADC with *EGFR*− (C). TMB, tumor mutational burden; SUL, standardized uptake value corrected by lean body mass; ADC, adenocarcinoma; *EGFR*, epidermal growth factor receptor.

Figure 2 demonstrates the multivariate linear regression analyses for the independent predictors of TMB (lg). Age (*β* = 0.212, *p* = 0.010), ADC (*β* = −0.184, *p* = 0.043), *EGFR*+ (*β* = −0.269, *p* = 0.005), and SUL_peak_ (*β* = 0.345, *p* < 0.001) were independent predictors for NSCLC; age (*β* = 0.243, *p* = 0.012), *EGFR*+ (*β* = −0.241, *p* = 0.010), high CEA (*β* = 0.201, *p* = 0.044), and SUL_peak_ (*β* = 0.355, *p* = 0.001) were independent predictors for ADC; SUL_peak_ (*β* = 0.342, *p* = 0.011) was an independent predictor for ADC with *EGFR*+; and age (*β* = 0.411, *p* = 0.003), high CEA (*β* = 0.424, *p* = 0.002), and SUL_peak_ (*β* = 0.439, *p* = 0.002) were independent predictors for ADC with *EGFR*−. SUL_peak_ was an independent predictor of TMB (lg) for all the above categories (NSCLC, ADC, ADC with *EGFR*+, and ADC with *EGFR*−). Figures 3 and 4 show the significant positive linear correlations between SUL_peak_ and TMB (lg) for all the above categories, and no significant correlation was observed for SCC.

## 4 DISCUSSION

Correlations between MPs, STMs, and TMB remain poorly understood. This study revealed different findings between ADC and SCC. For ADC, pretreated MPs and STMs were generally higher in the TMB‐high group than in the non‐TMB‐high group; all MPs showed significant positive linear correlations with TMB (lg), and SUL_peak_ was an independent predictor of TMB (lg), including subgroups of ADC with *EGFR*+ and *EGFR*−. However, no significant correlation was observed for SCC. We identified that MPs and STMs can predict the TMB level for patients with ADC, and may serve as potential substitutes for TMB with increased value and easy implementation in guiding immunotherapy through noninvasive methods.

The present study had the following improvements over previous studies: (a) subgroup analysis of the *EGFR* mutation was performed for the first time, as we believed it was important that patients with *EGFR*− were the target candidates for immunotherapy; (b) correlations between SUL parameters and TMB were analyzed for the first time. SUL parameters eliminate the influence of adipose tissue compared to SUV parameters. PERCIST 1.0 also recommends SUL_peak_ as the most important quantitative indicator of therapeutic response for immunotherapy; (c) CA125 and SCC‐Ag were studied for the first time, which enriched the blood‐based biomarkers; (d) TMB‐high was defined as TMB ≥10 mutations/Mb as it may be the optimal and universal cutoff to guide clinical applications; and (e) we set a 1‐month interval between PET/CT scan, STM test, and biopsy/surgery to ensure an acceptable temporal correlation. A longer time lapse may result in a poor correlation.

This study demonstrated that TMB‐high in NSCLC was associated with male, smoker, and SCC, which was consistent with the result of previous studies.^11^, ^19^, ^20^ Correlations of MPs and STMs with TMB only existed in ADC, and no significant correlation was observed for SCC. The reasons for the different results between ADC and SCC remain unclear. In our previous publication, we reported that baseline SUL_max_ and SUL_peak_ were positively correlated with the degree of pathological regression to neoadjuvant immunotherapy in NSCLC (SUL_max_, *p* = 0.036; SUL_peak_, *p* = 0.058), and a large majority of the patients (80.6%, 29/36) had SCC.^4^ Therefore, we speculated that there may be links between MPs and immunotherapy biomarkers in SCC. However, no significant correlation was observed for SCC in this study. One reason could be the limited number of patients. Further studies involving more patients are required. Another possibility is that MPs correlate with programmed death ligand 1 (PD‐L1) expression instead of TMB in SCC. Previous studies have reported a significant positive correlation between SUV_max_ and PD‐L1 expression in SCC.^21^, ^22^ It has been proven that PD‐L1 expression is independent of TMB, and both can independently predict immunotherapy efficacy.^23^ The predictive effect of PD‐L1 expression and TMB, alone or in combination, and their correlations with tumor metabolism deserve further study.

A negative correlation was observed between *EGFR*+ and TMB, which may explain why *EGFR*+ patients had a poor response to ICIs and even underwent hyperprogression.^24^, ^25^ Among the 53 ADC patients with *EGFR*+ in this study, only one patient had TMB‐high (TMB, 15 mutations/Mb), which was contradictory to the result of the negative correlation. However, the patient also had *KRAS*+. *KRAS*+ was found to be a predictor of TMB‐high and positively correlated with TMB in the univariate linear analysis. Previous studies have also reported that patients with *KRAS*+ had better immunotherapy efficacy.^26^ Therefore, *KRAS*+ may be an important predictor of TMB‐high, which needs further verification.

We found that all MPs (SUL_peak_, SUL_max_, SUL_mean_, MTV, and TLG) were significantly higher in the TMB‐high group than in the non‐TMB‐high group, and all MPs presented significant positive correlations with TMB. However, the exact mechanism between FDG uptake and TMB remains unclear. The mechanism of FDG uptake involves glucose metabolism, angiogenesis, hypoxia, and the mammalian target of rapamycin (mTOR) signaling pathway.^27^ A previous study showed that carbohydrate metabolism had a positive correlation with TMB,^28^ and another study showed that mutations of the mTOR pathway were associated with increased TMB.^29^ Thus, the correlation between high MPs and high TMB may reflect increased glucose metabolism and activation of the mTOR pathway. Another alternative explanation for the correlation may be based on an innate immune response triggered by tumors with higher TMB. FDG is actively entrapped in the increased infiltration of immune effector cells such as tumor‐infiltrating lymphocytes and tumor‐associated macrophages.^30^ In the presence of the activated mTOR pathway, pathway enrichment was involved in antitumor immunity, enhanced antigen presentation, and increased infiltration of immune effector cells.^29^ Moreover, tumor aggressiveness may be another intrinsic link between MPs and TMB. It is generally believed that tumors with high FDG uptake have a higher likelihood of aggressiveness,^31^ and the phenotypic features related to aggressiveness may become more prominent as the genetic mutation rate increases.^32^

The molecular pathogenesis of lung cancer involves various oncogenes, tumor suppressor genes, signaling pathway components, and other cellular processes.^33^ These molecular processes lead to the release of various mutated or overexpressed proteins associated with lung cancer into body fluids, known as STMs, which are potential noninvasive biomarkers for diagnosis, monitoring, and prognosis assessment.^34^ The correlation between STMs and TMB remains poorly understood. Ono et al. reported that CEA level was an independent predictor of TMB.^12^ The present study also found that high CEA and CA125 levels were independent predictors of TMB‐high. The reappearance of CEA in patients with cancer indicates that certain genes are reactivated by the malignant transformation of cells. CEA can serve as an ideal tumor‐associated antigen for inducing effective tumor immunity.^7^

The present study had several limitations. First, this was a preliminary, exploratory, and single‐center retrospective study with a small number of patients, although approximately 4 years of data were included. Second, we only studied *EGFR* and *KRAS* mutations. Other driver gene mutations were not included because of the limited number of patients. Third, not all patients had available STM data. Fourth, the spatial heterogeneity of tumors might influence TMB detection. Finally, exploring the direct relationship between MPs, STMs, and immunotherapy response is a future step. Therefore, prospective studies are necessary to validate our findings.

## 5 CONCLUSION

MPs and STMs can predict the TMB level for patients with ADC, and may serve as potential substitutes for TMB with increased value and easy implementation in guiding immunotherapy through noninvasive methods. We highlight the findings for ADC patients with *EGFR*−, as they are the target candidates for immunotherapy.

## AUTHOR CONTRIBUTIONS

**Qian Zhang:** Data curation (equal); formal analysis (equal); methodology (equal); resources (equal); software (equal); visualization (equal); writing – original draft (lead). **Xiuli Tao:** Conceptualization (equal); data curation (equal); formal analysis (equal); funding acquisition (lead); investigation (equal); methodology (equal); project administration (equal); supervision (equal); writing – review and editing (equal). **Pei Yuan:** Data curation (equal); formal analysis (equal); methodology (equal); resources (equal); software (equal). **Zewei Zhang:** Data curation (equal); formal analysis (equal); methodology (equal); resources (equal); software (equal); visualization (equal). **Jianming Ying:** Formal analysis (supporting); methodology (supporting); resources (supporting); software (supporting). **Lei Guo:** Formal analysis (supporting); methodology (supporting); resources (supporting); software (supporting). **Ning Li:** Formal analysis (supporting); methodology (supporting); resources (supporting); software (supporting). **Shuhang Wang:** Formal analysis (supporting); methodology (supporting); resources (supporting); software (supporting). **Jing Li:** Data curation (supporting); formal analysis (supporting); methodology (supporting); resources (supporting); software (supporting). **Ying Liu:** Data curation (supporting); formal analysis (supporting); methodology (supporting); resources (supporting); software (supporting). **wei guo:** Data curation (supporting); formal analysis (supporting); methodology (supporting); resources (supporting); software (supporting). **Shijun Zhao:** Conceptualization (equal); methodology (equal); project administration (equal); supervision (equal); writing – review and editing (equal). **Ning Wu:** Conceptualization (equal); data curation (equal); funding acquisition (supporting); project administration (lead); supervision (lead); writing – review and editing (lead).

## CONFLICT OF INTEREST STATEMENT

The authors declare that they have no conflicts of interest.

## ETHICS STATEMENT

The study was conducted according to the guidelines of the Declaration of Helsinki and approved by the Institutional Review Board of the Institutional Ethics Committee of the Cancer Hospital, Chinese Academy of Medical Sciences. The requirement for informed consent was waived because of the retrospective and anonymous nature of the study.

## Data Availability

The datasets used and analyzed during the current study are available from the corresponding author upon reasonable request.
